# Gene- and Disease-Based Expansion of the Knowledge on Inborn Errors of Immunity

**DOI:** 10.3389/fimmu.2019.02475

**Published:** 2019-10-21

**Authors:** Lyubov E. Salnikova, Ekaterina V. Chernyshova, Lyudmila A. Anastasevich, Sergey S. Larin

**Affiliations:** ^1^The Laboratory of Ecological Genetics, Vavilov Institute of General Genetics, Russian Academy of Sciences, Moscow, Russia; ^2^The Laboratory of Molecular Immunology, Rogachev National Research Center of Pediatric Hematology, Oncology and Immunology, Moscow, Russia; ^3^The Laboratory of Clinical Pathophysiology of Critical Conditions, Federal Research and Clinical Center of Intensive Care Medicine and Rehabilitology, Moscow, Russia

**Keywords:** international union of immunological societies, inborn errors of immunity, OMIM, ORPHANET, human phenotype ontology, clinical signs and symptoms, pleiotropy, gene-disease association

## Abstract

The recent report of the International Union of Immunological Societies (IUIS) has provided the categorized list of 354 inborn errors of immunity. We performed a systematic analysis of genes and diseases from the IUIS report with the use of the OMIM, ORPHANET, and HPO resources. To measure phenotypic similarity we applied the Jaccard/Tanimoto (J/T) coefficient for HPO terms and top-level categories. Low J/T coefficients for HPO terms for OMIM or ORPHANET disease pairs associated with the same genes indicated high pleiotropy of these genes. Gene ORGANizer enrichment analysis demonstrated that gene sets related to HPO top-level categories were most often enriched in immune, lymphatic, and corresponding body systems (for example, genes from the category “Cardiovascular” were enriched in cardiovascular system). We presented available data on frequent and very frequent clinical signs and symptoms in inborn errors of immunity. With the use of DisGeNET, we generated the list of 25 IUIS/OMIM diseases with two or more relatively high score gene-disease associations, found for unrelated genes and/or for clusters of genes coding for interacting proteins. Our study showed the enrichment of gene sets related to several IUIS categories with neoplastic and autoimmune diseases from the GWAS Catalog and reported individual genes with phenotypic overlap between inborn errors of immunity and GWAS diseases/traits. We concluded that genetic background may play a role in phenotypic diversity of inborn errors of immunity.

## Introduction

Rare diseases, being individually rare, altogether affect millions of people. Different estimates exist: rare diseases affect 10% of Americans (1) or 6–8% of Europeans (in total, more than 30 million citizens) (2). The prevalence of primary immunodeficiencies (PIDs) is reported at about 1:2,000 live births (3). Patients with rare diseases encounter with diagnostic difficulties. In the report produced by EURORDIS in 2007, 40% of rare disease patients first received an erroneous diagnosis, of which 25% had to wait for the correct diagnosis between 5 and 30 years. The patient or family was not informed on the genetic origin of the disease in 25% of cases (4). Since this publication, an enormous progress has been achieved in the genetic characterization of known diseases, in the search for new phenotypes and in the progress in rare disease differential diagnosis, however, the problem continues to be relevant. With the information explosion, both new opportunities and challenges constantly arise. The recent report of the International Union of Immunological Societies (IUIS) on inborn errors of immunity (5) has provided the categorized list of 354 inborn errors of immunity (as of 2017). Preliminary estimates indicate that in the next decade the number of PIDs may be as high as 3000 (6). As information accelerates, there is a constant need for the development of resources, algorithms and approaches to capture, process and analyze all this information.

With the manifold possible manifestations of inborn errors of immunity, careful characterization of gene-phenotype relationships is clinically relevant. Detailed reviews on rare individual disorders are available for many PIDs, however, the complex analysis of these disorders with the use of modern resources might shed light on some regularities in gene-disease interactions. The IUIS report may serve as a starting point for different types of in depth analysis of genes, diseases, and categories of inborn errors of immunity.

The study was designed to perform a systematic analysis of genes and diseases from the IUIS report. We set out to characterize gene-disease categories and the whole gene-disease set with the use of several resources widely known in the rare disease community: OMIM (7), ORPHANET (8), and HPO (9). We aimed to provide some new results for gene-disease associations available from public data and to attract the attention of researchers to the possible influence of common genetic variants on the phenotypic variability of inborn errors of immunity.

## Materials and Methods

### The OMIM, ORPHANET, and HPO Resources

OMIM and ORPHANET are the two largest rare disease public resources. OMIM is a continuously updated catalog of human genes and genetic phenotypes of all known Mendelian disorders (7). OMIM content is generated with the use of published peer-reviewed biomedical literature. It includes over 25,000 entries with approximately 9,000 of them related to phenotypes (as of 28 June 2019). OMIM information is provided as free text; genes and phenotypes are described in separate entries and have unique digit identifiers (MIM numbers). Orphanet is a European initiative aimed at collecting expert-vetted data on rare diseases and their associated genes from multiple information resources (8). ORPHANET provides data for 21,566 diseases with all available synonyms (as of 18 February 2019). ORPHANET contains rare diseases ontology (ORDO) with a systematic vocabulary for rare diseases, relationships between diseases, genes associated with these diseases and other relevant features. The Human Phenotype Ontology (HPO) is organized as a standardized vocabulary integrating data on phenotypic abnormalities from literature and rare disease resources (9). It currently contains over 13,000 terms and over 156,000 annotations to hereditary diseases (release date June 16, 2019). The HPO data may be queried for genes, HPO terms and diseases (separately for OMIM diseases and ORPHANET diseases).

We searched OMIM and ORPHANET for genes from the IUIS report and assessed the compliance of associated diseases and phenotypes with those presented in the IUIS report. The HPO database was first queried for genes and then for associated HPO terms and diseases (separately for OMIM diseases and ORPHANET diseases). For further analysis we exported disease-associated HPO term names together with their top-level categories.

### The DisGeNET Resource

DisGeNET is one of the largest and comprehensive repositories of human gene-disease associations (10). It comprises data on 628,685 gene-disease associations between 17,549 genes and 24,166 diseases/traits (as of January 2019). DisGeNET includes associations for Mendelian, complex, environmental and rare diseases, and disease-related traits.

We queried DisGeNET separately for genes and OMIM and/or ORPHANET diseases for summaries of all gene-disease and variant-disease associations, which included, in particular, original metrics describing scores for these associations, disease specificity and pleiotropy indexes. To improve reliability, we mainly focused on records with gene-disease association score ≥ 0.3. The metrics were used to evaluate genotype-phenotype relationships.

### Jaccard/Tanimoto Similarity Test

The Jaccard/Tanimoto coefficient is the ratio of intersection to union used to measure similarity between binary data (11). It estimates the ratio of the numbers of the elements in the intersecting and union sets as the measure of similarity (it equals to zero in the absence of intersecting elements and equals to one when all elements overlap). The Jaccard/Tanimoto coefficient was calculated by the equation

$$\begin{array}{l} T=\;\frac{Nc}{Na+Nb-Nc} \end{array}$$

(*Na*- number of elements in set A; *Nb*- number of elements in set B; *Nc*- number of elements in intersecting set) for pairwise testing of means. Jaccard/Tanimoto coefficients significance was calculated with a CRAN R package (12). To correct for multiplicity we applied Benjamini and Hochberg false discovery rate (FDR) with a threshold < 0.05.

### Gene Set Enrichment Analysis

Enrichment analysis is a leading computational method for analyzing gene sets in the context of prior biological knowledge. We implemented a phenotype-based tool Gene ORGANizer for the enrichment analysis focused on body systems (13). Gene ORGANizer comprises HPO and DisGeNET phenotype associations for more than 7,000 genes. For the IUIS/OMIM diseases with more than one high-score gene-disease association in the DisGeNET database we applied enrichment for protein-protein interactions (PPI) conducted with the Search Tool for the Retrieval of Interacting Genes (STRING) database (14). We analyzed human PPI dataset taking the combined score > 0.4 as the cut-off criterion. Overlap with gene set library from the NHGRI-EBI GWAS Catalog (GWAS Catalog 2019) was assessed with the Enrichr tool (15). We set Benjamini and Hochberg false discovery rate (FDR) to 5% (corrected for multiplicity *P* value < 0.05).

## Results

### Disease Matching in the IUIS, OMIM, and ORPHANET Databases

We defined a total of 331 unique genes (further IUIS genes) linked to 300 unique diseases (further IUIS diseases) from eight of nine IUIS categories (5). One category “Phenocopies of inborn errors of immunity,” which includes non-inborn errors of immunity was not covered by our analysis. For disease matching, we used gene and disease names and associated features from the IUIS tables, and the OMIM and ORPHANET information. ORPHANET differs from OMIM and IUIS by the level of granularity. In the ORPHANET database, individual gene-linked disorders may be related to parent diseases associated with a group of genes. For example, seven OMIM Aicardi-Goutieres syndrome phenotypes (Aicardi-Goutieres syndrome 1–7) are merged into one ORPHANET disease “Aicardi-Goutières syndrome.” OMIM-ORPHANET matching was facilitated by the fact, that ORPHANET diseases have a link to the relevant phenotype in the OMIM database. Although the IUIS report also contains OMIM numbers for gene-disease pairs, these numbers are often related to gene, not phenotype descriptors. One gene may be associated with several clinical phenotypes indicated by Phenotype MIM number. For example, the IUIS disease “β actin deficiency” (gene *ACTB*) corresponds to MIM phenotypes “Baraitser-Winter syndrome 1” (243310) and “Dystonia, juvenile-onset” (607371), or “Adenosine deaminase (ADA) deficiency” (gene *ADA*) in the IUIS Report is related to “Omenn Syndrome” (603554) and “Severe Combined Immunodeficiency, Autosomal Recessive, T Cell-negative, B Cell-negative, NK Cell-negative, Due To Adenosine Deaminase Deficiency” (102700). In some cases, the IUIS-OMIM disease matching was a judgment call based on the analysis of literature and OMIM full text descriptions. All IUIS gene-disease pairs were matched with corresponding OMIM and/or ORPHANET gene-disease pairs (in total 300 gene-disease pairs in one or both databases). Matching of the IUIS diseases provided 329 MIM phenotypes and 262 ORPHANET diseases (Supplementary Table 1, Sheet “IUIS_Diseases”). The IUIS genes were also associated with some other rare diseases (Supplementary Table 1, Sheet “non-IUIS_Diseases”).

### Representation and Characterization of the IUIS Diseases in the ORPHANET Database

**Figure 1:**
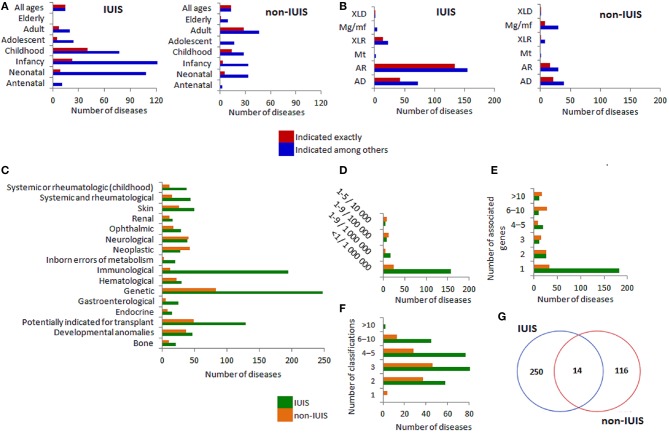
ORPHANET annotations for IUIS/non-IUIS diseases. The distribution of the diseases associated with the IUIS genes by **(A)** age of onset of the IUIS diseases (left) and non-IUIS diseases (right), **(B)** mode of inheritance for the IUIS diseases (left) and non-IUIS diseases (right), **(C)** ORPHANET classifications for rare diseases (classifications including 10 or more diseases are presented), **(D)** prevalence, **(E)** number of associated genes, **(F)** number of classifications per disease, **(G)** gene-centered Venn diagram for the IUIS- and non-IUIS diseases in the ORPHANET database. **(A,B)** Since many diseases were associated with more than one gene, we characterized found associations (Y-axis) as indicated among others (different genes are linked to several variants of disease manifestation) and indicated exactly (corresponds to the axis labels). AD, Autosomal dominant; AR, Autosomal recessive; Mt, Mitochondrial inheritance; XLR, X-linked recessive; Mg/mf, Multigenic/mulifactorial; XLD, X-linked dominant.

ORPHANET annotations for the diseases associated with the IUIS genes are presented in Figure 1. These annotations were generated using information from Supplementary Table 1. ORPHANET level of granularity determines the association of one disease with one or several genes. This feature is reflected in Figures 1A,B displaying age of onset and mode of inheritance of the IUIS and non-IUIS diseases as indicated exactly and indicated among others (for multigene associations). The IUIS diseases predominantly manifest in neonatal age, infancy and childhood, while non-IUIS diseases may manifest throughout life (Figure 1A). Autosomal recessive (AR) and autosomal dominant (AD) modes of inheritance are prevalent among the IUIS diseases. AR, AD, and muligenic/multifactorial modes of inheritance are similar in frequency among non-IUIS diseases (Figure 1B). As expected, such ORPHANET classifications as rare genetic diseases, rare immunological diseases and rare disorders potentially indicated for transplant are related to the vast majority of the IUIS diseases. Among non-IUIS diseases, neurological and neoplastic diseases are relatively numerous (Figure 1C). The most rare disease prevalence (< 1/1,000,000) is typical for the IUIS diseases and not strongly prevails among non-IUIS diseases (Figure 1D). In contrast to non-IUIS diseases, the IUIS diseases are mainly associated with a single gene (Figure 1F). The distribution of the number of ORPHANET classifications for the IUIS and non-IUIS diseases is reflected in Figure 1G. A small overlap between the IUIS and non-IUIS diseases (Figure 1G) is explained by the ORPHANET level of granularity.

Summarizing ORPHANET data, we may state that inborn errors of immunity were mainly represented by immunological diseases with autosomal recessive or autosomal dominant inheritance, very rare prevalence (< 1/1,000,000) and early age of disease manifestation. The majority of diseases were related to more than two classifications.

### HPO Terms and Top-Level Categories for the Genes Associated With Inborn Errors of Immunity

**Figure 2:**
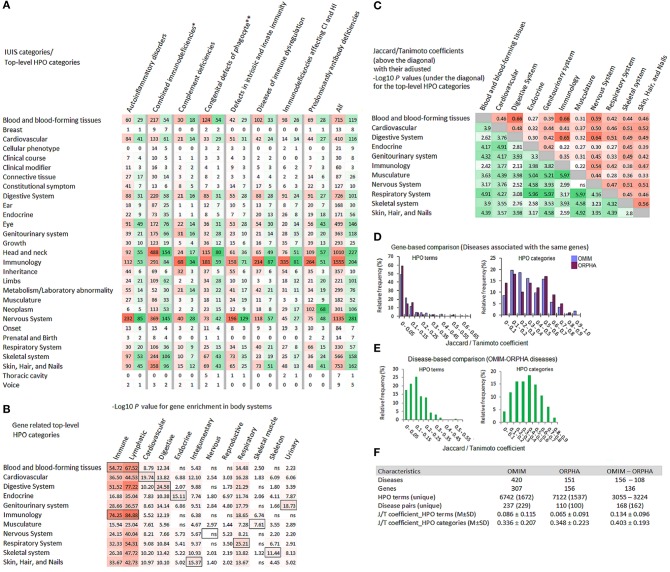
HPO annotations for the genes associated with inborn errors of immunity. **(A)** Multiple heat-map matrix for HPO terms related to top-level HPO categories (Y-axis) in the diseases grouped by the IUIS categories (X-axis). Since many HPO terms were associated with several genes, the data are presented as sum of their occurrences, i.e., the total number (red) and unique number (green) of HPO terms. For the IUIS categories all heat-maps are independent to each other. **(B)** Heat-map matrix for the results of Gene ORGANizer enrichment analysis. Gene lists related to top-level HPO categories (Y-axis) were analyzed for their overrepresentation in body systems (X-axis). FDR adjusted enrichment *P* values (threshold < 0.05) represented as –Log10 (threshold > 1.30) are plotted against HPO categories. Cells with *P* values for body systems matched with HPO categories are highlighted with black boundaries. **(C)** Heat-map matrix for the genetic similarity between selected top-level HPO categories. Jaccard/Tanimoto coefficient values are colored in red; their FDR adjusted *P* values represented as –Log10 are colored in green. **(D)** The Jaccard/ Tanimoto coefficient distribution for HPO terms and top-level HPO-categories for OMIM and ORPHANET diseases associated with the same genes. **(E)** The Jaccard/Tanimoto coefficient distribution for HPO terms and top-level HPO-categories for matched OMIM-ORPHANET disease pairs. **(F)** Summary statistics for the overlap measures. CI, cellular immunity; FDR, false discovery rate; HI, humoral immunity; J/T, Jaccard/Tanimoto; ns, non-significant; ORPHA, ORPHANET. ^*^Combined immunodeficiencies with associated or syndromic features; ^**^Congenital defects of phagocyte number or function.

The HPO data on phenotypic abnormalities, associated with the IUIS genes, are presented in Figure 2. In total, there were 10,506 HPO terms, among them, 2,229 terms were unique. Ten top-level HPO categories (blood and blood-forming tissues; cardiovascular; digestive system; eye; genitourinary system; head and neck; immunology; nervous system; skeletal system; skin, hair, and nails) included the largest numbers of HPO terms (in total, and unique terms) (Figure 2A). The distribution of the top ten HPO terms mapped to these HPO categories is presented in Supplementary Figure 1. The greatest number and variety of HPO terms is related to three top-level categories (nervous system, head and neck, and immunology) (Figure 2A). The enrichment analysis focused on 12 body systems showed that gene sets related to HPO categories were most often enriched in immune, lymphatic and corresponding body systems (for example, genes from the category “Cardiovascular” were enriched in cardiovascular system). The unexpected exclusion was “Nervous system” (Figure 2B). The genetic similarity between pairs of 11 HPO categories assessed by the Jaccard/Tanimoto coefficient was relatively high and significant (with one exception) (Figure 2C). The Jaccard/Tanimoto coefficient was used to measure the phenotypic similarity of diseases associated with the same gene. In the OMIM and ORPHANET databases, 99 and 43 genes related to ≥ 2 diseases were linked to 236 and 110 unique disease pairs, respectively. This gene-based approach revealed a weak similarity at the HPO term level and a rather high similarity at the HPO category level (Figure 2D). The Jaccared/Tanimoto coefficients were relatively high for the OMIM-ORPHANET matched disease pairs (*n* = 136) (Figure 2E). The Jaccared/Tanimoto coefficients for the HPO terms and categories are presented in Figure 2F. At the level of HPO terms, significant differences were revealed between the Jaccared/Tanimoto coefficients related to OMIM (gene-based comparison) and OMIM-ORPHANET (disease-based comparison) (Mann-Whitney test: *P* = 3.185E−11) and ORPHANET (gene-based comparison) and OMIM-ORPHANET (disease-based comparison) (Mann-Whitney test: *P* = 5.98E−12). At the level of HPO categories, the differences were less pronounced (OMIM and OMIM-ORPHANET *P* = 5.75E-04; ORPHANET; and OMIM-ORPHANET *P* = 6.10E-02, non-significant). Despite the differences in the OMIM and ORPHANET hierarchy for disease classification, the comparison yielded higher Jaccared/Tanimoto coefficients for the matched OMIM-ORPHANET diseases than for the disease pairs associated with the same genes.

Briefly, this part of our study presents an overview of HPO terms and their top-level categories associated with the IUIS genes. The enrichment analysis via Gene ORGANizer provided information on the overrepresentation of HPO terms at the level of 12 body systems assessed in a genomic context. Different HPO categories were represented by highly overlapped gene spectra. The analysis of phenotypic similarity measured by the Jaccard/Tanimoto coefficient showed that although diseases associated with the same genes are very different in their clinical signs and symptoms, the latter are related to the limited number of HPO top-level categories, i.e., mainly affect certain body systems.

### Frequent and Very Frequent Clinical Signs and Symptoms in the Inborn Errors of Immunity

**Figure 3:**
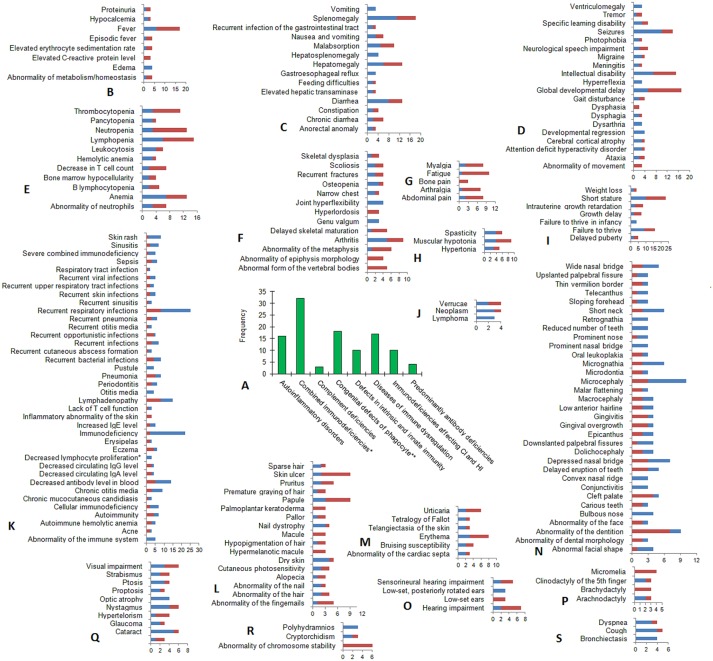
Frequent and very frequent clinical signs and symptoms in diseases matched between IUIS and ORPHANET. **(A)** The distribution of diseases within the IUIS categories (for 110 diseases with available annotations on symptom frequency). **(B–S)** The frequency of occurrence (X-axis) of frequent (dark red) and very frequent (blue) clinical signs and symptoms (Y-axis) is mapped to HPO term names within top-level HPO categories: **(B)** Metabolism/Laboratory abnormality, **(C)** Digestive System, **(D)** Nervous System, **(E)** Blood and blood-forming tissues, **(F)** Skeletal system, **(G)** Constitutional Symptom, **(H)** Musculature, **(I)** Growth, **(J)** Neoplasm, **(K)** Immunology, **(L)** Skin, Hair, and Nails, **(M)** Cardiovascular, **(N)** Head and neck, **(O)** Ear, **(P)** Limbs, **(Q)** Eye, **(R)** Miscellaneous: Genitourinary system (Cryptorchidism), Prenatal and Birth (Polyhydramnios) and Cellular phenotype (Abnormality of chromosome stability), (S) Respiratory System. *Combined immunodeficiencies with associated or syndromic features; **Congenital defects of phagocyte number or function. CI, cellular immunity; HI, humoral immunity.

ORPHANET provides data on symptom frequency. Currently, these data are available for 110 inborn errors of immunity. The list of 110 diseases is given in Supplementary Table 2. Among the disease categories, the largest number of annotated diseases is related to “Combined immunodeficiencies with associated or syndromic features” (Figure 3A). All frequent and very frequent clinical signs and symptoms occurring in three or more diseases are provided in Figures 3B–S. Frequent symptoms occur in 79%−30% of patients; very frequent symptoms are typical for 80%−99% of patients. The greatest symptom variety is revealed for the following top-level HPO categories: “immunology,” *n* = 38; “head and neck,” *n* = 33; and “nervous system,” *n* = 20.

### Gene-Disease Association Score and IUIS/OMIM Diseases With More Than One High-Score Association

The DisGeNET resource collects data on gene-disease associations for a wide range of diseases, including rare diseases. It ranges gene-disease associations by score, which depends on the number and type of sources and the number of publications in support of the association. We divided all gene-disease associations found for inborn errors of immunity in the DisGeNET resource (in total, 179 diseases) into three groups: S_1_, with gene-disease association score ≥ 0.3; S_2_, with gene-disease association score 0.1– 0.29; S_3_ with gene-disease association score < 0.1. The full list of the genetic associations and their scores is presented in Supplementary Table 3. Inborn errors of immunity (*n* = 25) with more than one high-score association in the DisGeNET database are listed in Table 1. The PPI enrichment analysis showed that these high score gene-disease associations were found for both, clusters of encoded interacting proteins and unrelated proteins. The IUIS/OMIM gene-disease associations usually have the highest scores; however, other disease associations may also matter.

**Table 1:** IUIS/OMIM diseases with more than one high-score gene-disease association in the DisGeNET database.

**Disease**	**#**	**Gene symbols (S)**	**PPI enrichment *P* value**
Aicardi-Goutieres syndrome 2	6	***RNASEH2B*** **(0.6)**–*TREX1* (0.5)–*ADAR* (0.3)–*IFIH1* (0.3)–*USP18* (0.3)–*SAMHD1* (0.3)	8.44E−15
Aplastic anemia	7	^*^TERT (0.69)–*IFNG* (0.6)–*PRF1* (0.4)–*CSF3* (0.37)–*CSF2* (0.34)–*SBDS* (0.32); *NBN* (0.42)	8.94E−05
Autoimmune lymphoproliferative syndrome	6	***FAS*** **(1.0)**–*FASLG* (0.93)–*CASP10* (0.34)–*PRKCD* (0.31); *NRAS* (0.32)–*CASP8* (0.3)	1.18E−05
Autoimmune lymphoproliferative syndrome, type IB	2	***FASLG*** **(0.4)**–*FAS* (0.3)	3.01E−02
Bare lymphocyte syndrome, type I	4	*TAP2* (0.51)–*TAPBP* (0.51)–***TAP1*** **(0.5)**–*B2M* (0.3)	2.67E−12
Bare lymphocyte syndrome, type II, complementation group A	3	***CIITA*** **(0.4)**–*RFX5* (0.3)–*RFXANK* (0.3)	9.06E−07
Bare lymphocyte syndrome, type II, complementation group C	3	***RFX5*** **(0.3)**–*CIITA* (0.3)–*RFXANK* (0.3)	9.06E−07
Bare lymphocyte syndrome, type II, complementation group D	3	^**^CIITA (0.3)–*RFX5* (0.3)–*RFXANK* (0.3)	9.06E-07
Bare lymphocyte syndrome, type II, complementation group E	3	***RFX5*** **(0.4)**–*CIITA* (0.3)–*RFXANK* (0.3)	9.06E−07
Bloom syndrome	2	***BLM*** **(1.0)**; *UNG* (0.35)	1.0
Bone marrow failure syndrome 2	4	***ERCC6L2*** **(0.6)**; *CSF2* (0.3); *SRP72* (0.3); *DNAJC21* (0.3)	1.0
CHARGE syndrome	2	***CHD7*** **(1.0)**; *SEMA3E* (0.6)	1.0
Cystic fibrosis	9	***CFTR*** **(1.0)**–*SCNN1B* (0.6)–*SCNN1A* (0.37)–*SCNN1G* (0.36)– *STX1A* (0.31); *TGFB1* (0.6); *DCTN4* (0.52); *CLCA4* (0.34); *TNFRSF1A* (0.31)	2.76E-07
DiGeorge syndrome	16	***TBX1*** **(0.9)**–*COMT* (0.6)–*CRKL* (0.51)–*FGF8* (0.5)–*HIRA* (0.4)–*DGCR* (0.4)–*UFD1* (0.33)–*DGCR6* (0.33)–*DGCR2* (0.32)–*GP1BB* (0.31)–*ARVCF* (0.3)–*ESS2* (0.3);*DGCR8* (0.31); *JMJD1C* (0.3); *SEC24C* (0.3); *RREB1* (0.3)	<1.0E−16
Immunodeficiency 44	2	***STAT2*** **(0.6)**–*IFNAR2* (0.3)	1.16E−02
Leukemia, acute myeloid, susceptibility to	4	^***^SETD2 (0.3)–*CEBPA* (0.3)–*SETBP1* (0.3)–*JAK2* (0.3)	1.17E−05
LIG4 syndrome	2	***LIG4*** **(0.99)**–*XRCC4* (0.3)	1.70E−02
Muckle-Wells syndrome	4	***NLRP3*** **(0.8)**–*NLRC4* (0.3); *MME* (0.3); *PLCG2* (0.3)	0.12
Myopathy, tubular aggregate, 1	2	***STIM1*** **(0.3)**–*CASQ1* (0.3)	3.37E−03
Omenn syndrome	15	***RAG2*** **(0.8)**–***RAG1*** **(0.6)**–***DCLRE1C*** **(0.55)**–***IL7R*** **(0.33)**–*IL2RG* (0.32)–***ADA*** **(0.31)**–*CD3E* (0.31)–*ZAP70* (0.31)–*CD3D* (0.3)–*TFRC* (0.3)–*AK2* (0.3)– *LIG4* (0.3)– *JAK3* (0.3); *CHD7* (0.31); ^****^RMRP (0.3)	<1.0E−16
Osteopetrosis, autosomal recessive 6	3	***PLEKHM1*** **(0.8)**–*CLCN7* (0.3)–*TCIRG1* (0.3)	9.14E−08
Specific granule deficiency	2	***CEBPE*** **(0.61);** *SMARCD2* (0.6)	1.0
Specific granule deficiency 2	2	***SMARCD2*** **(0.4);** *CEBPE* (0.3)	1.0
Tetralogy of fallot	16	*ZFPM2* (0.98)–*NKX2-5* (0.96)–*GATA4* (0.8)–*JAG1* (0.8)–*GATA6* (0.73)–*GDF1* (0.71)–*GJA5* (0.62)–*CITED2* (0.6)–***TBX1*** **(0.46)**–*GATA5* (0.42)–*NKX2-6* (0.4)–*FOXC2* (0.4)– *HAND2* (0.31)–*FOXC1* (0.3)–*FLT4* (0.3)–*FOXH1* (0.3)	<1.0E−16
Wiskott-Aldrich syndrome	2	***WAS*** **(1.0)**–*WIPF1* (0.6)	1.70E−02

*S, Gene-disease association score (S ≥ 0.3); PPI, Protein-protein interaction*.

Genes from the IUIS or OMIM resource are in bold. Genes related to the same cluster are connected with a dash. Genes not related to the same cluster are separated by a semicolon. An asterisk indicates that gene associated with the disease in the IUIS or OMIM resource was not found for the same gene-disease association in the DisGeNET resource (^*^TERC, ^**^RFXAP, ^***^GATA2) or STRING (^****^RMRP).

Given these results, the most prominent gene candidates for inborn errors of immunity with unidentified genetic abnormalities in known genes are often represented by other genes involved in the PPI network.

### Specificity and Pleiotropy of Genes Associated With Inborn Errors of Immunity

**Figure 4:**
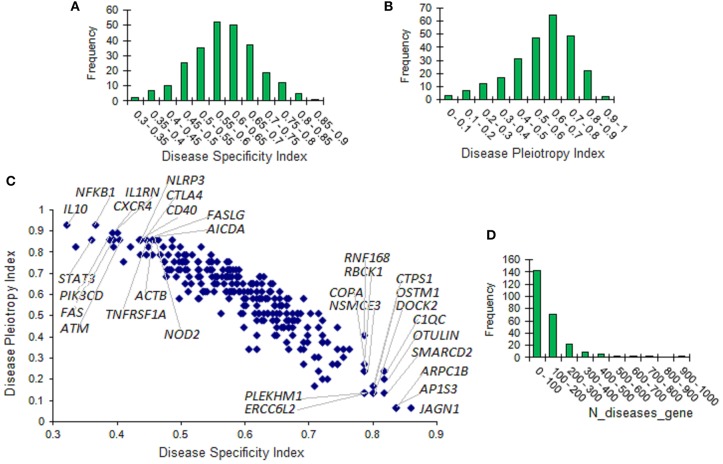
Disease specificity and disease pleiotropy indexes of genes associated with inborn errors of immunity. **(A)** The distribution of the disease specificity index (DSI). **(B)** The distribution of the disease pleiotropy index (DPI). **(C)** Scatterplot of DSI and DPI. Ten top values are presented for DSI (15 genes) and DPI (16 genes). **(D)** The distribution of the number of associated diseases.

DisGeNET comprises 6,780 disease associations for the IUIS genes with in total 36,169 gene-disease association pairs. To address the item on gene specificity and pleiotropy, we used DisGeNET metrics, which include, in particular, the Disease Sensitivity Index (DSI) and the Disease Pleiotropy index (DPI). DSI is inversely proportional to the number of diseases associated with the corresponding gene. DPI is calculated taking into account whether the diseases associated with the gene are similar or completely different and refer to different classes of diseases. DSI and DPI were available for 255 IUIS genes. The distribution of DSI and DPI for these genes is presented in Figures 4A,B. The scatterplot displaying the relationship between DSI and DPI is produced in Figure 4C. Ten top values are indicated for DSI (15 genes) and DPI (16 genes). The distribution of the number of associated diseases is shown in Figure 4D. Large numbers of associated diseases per gene are explained by the fact that all associations are taken into account regardless of their scores.

Besides gene-disease associations, DisGeNET also provides information on variant-disease associations. From the total of 11,884 variants in the IUIS genes, 5,084 variants had data on allele frequency. The minimum alternative allele frequency (AF) was 3.18E−05; 2,999 variants had AF > 0.05. Variant-disease association scores in the range of 0.65–1.0 were registered for 2,657 of 5,084 variants. These data are presented in Supplementary Figure 2.

### Phenotypic and Genetic Overlap Between Inborn Errors of Immunity and Complex Traits

Given that all IUIS genes are associated with several or many diseases, and a lot of these associations have been found for common genetic variants, we searched the NHGRI-EBI GWAS Catalog (16) for the genes of inborn errors of immunity in order to see if there is phenotypic and genetic overlap between inborn errors of immunity and complex traits. A comparison of HPO terms and GWAS mapped traits yielded 35 genes with overlapping phenotypes, among them autoimmune and neoplastic phenotypes were highly represented (Table 2). The largest number of associations with neoplasms has been registered for the *TERT* gene (22 phenotypes).

**Table 2:** Overlap between HPO terms and GWAS mapped traits for the genes of inborn errors of immunity.

**Human Phenotype (HP)**	**Gene**	**GWAS mapped trait**
**Autoinflammatory disorders**		
Psoriasiform dermatitis (HP:0003765)	*CARD14*	Psoriasis
Diabetes mellitus (HP:0000819) Hypothyroidism (HP:0000821)	^*^IFIH1	Type I diabetes mellitus Hypothyroidism
Ulcerative colitis (HP:0100279)	*PLCG2*	Ulcerative colitis
Diabetes mellitus (HP:0000819)	*SAMHD1*	Diabetic nephropathy, type II diabetes mellitus
**Combined immunodeficiencies with associated or syndromic features**		
Neoplasm (HP:0002664)	*ATM*	Melanoma, prostate carcinoma, renal cell carcinoma, uterine fibroid
Conductive hearing impairment (HP:0000405)	*CCBE1*	Age-related hearing impairment
Depressivity (HP:0000716)	*CHD7*	Unipolar depression
Recurrent respiratory infections (HP:0002205)	*NFKBIA*	Respiratory system disease
Neoplasm (HP:0002664) Colitis (HP:0002583) Cough (HP:0012735), Honeycomb lung (HP:0025175), Pulmonary fibrosis (HP:0002206), Pulmonary insufficiency (HP:0010444), Recurrent respiratory infections (HP:0002205)	*RTEL1*	Central nervous system cancer, glioblastoma multiforme, glioma Ulcerative colitis Respiratory system disease
Scoliosis (HP:0002650)	*SEMA3E*	Adolescent idiopathic scoliosis
Emphysema (HP:0002097)	*SPINK5*	Response to bronchodilator, chronic obstructive pulmonary disease, FEV/FEC ratio
Eczema (HP:0000964) Diabetes mellitus (HP:0000819)	*STAT3*	Atopic eczema Type II diabetes mellitus
Honeycomb lung (HP:0025175)	*STN1*	Interstitial lung disease
Lymphoma (HP:0002665)	*TERC*	Hodgkins lymphoma, multiple myeloma, chronic lymphocytic leukemia
Neoplasm (HP:0002664) Neoplasm of the pancreas (HP:0002894) Neoplasm of the breast (HP:0100013) Melanoma (HP:0002861) Honeycomb lung (HP:0025175)	*TERT*	Breast carcinoma, estrogen-receptor negative breast cancer, triple-negative breast cancer, central nervous system cancer, glioblastoma multiforme, glioma, nasopharyngeal neoplasm, lung carcinoma, lung adenocarcinoma, squamous cell lung carcinoma, thyroid carcinoma, colorectal cancer, testicular carcinoma, testicular germ cell tumor, prostate carcinoma, ovarian carcinoma, ovarian endometrioid carcinoma, ovarian serous carcinoma, malignant epithelial tumor of ovary, chronic lymphocytic leukemia, pancreatic carcinoma, melanomaIdiopathic pulmonary fibrosis
Short stature (HP:0004322)	*ZBTB24*	Body height
**Complement deficiencies**		
Systemic lupus erythematosus(HP:0002725)	*C2*	Systemic lupus erythematosus
Macular degeneration (HP:0000608)	*CFHR1*	Atrophic macular degeneration, age-related macular degeneration, wet macular degeneration
Deep venous thrombosis (HP:0002625)	*THBD*	Venous thromboembolism
**Congenital defects of phagocyte number or function**		
Eczema (HP:0000964)	*NCF4*	Atopic eczema
**Defects in intrinsic and innate immunity**		
Stage 5 chronic kidney disease (HP:0003774), focal segmental glomerulosclerosis (HP:0000097)	*APOL1*	Chronic kidney disease
Amyotrophic lateral sclerosis (HP:0007354)	*TBK1*	Amyotrophic lateral sclerosis
**Diseases of immune dysregulation**		
Alopecia (HP:0001596) Autoimmunity (HP:0002960)	*CTLA4*	Alopecia areata Autoimmune disease
Alopecia (HP:0001596) Autoimmunity (HP:0002960) Eczema (HP:0000964) Diabetes mellitus (HP:0000819) Recurrent respiratory infections (HP:0002205) Juvenile rheumatoid arthritis (HP:0005681), Polyarticular arthritis (HP:0005764)	*IL2RA*	Alopecia areata Autoimmune disease Atopic eczema Type I diabetes mellitus Respiratory system disease Systemic juvenile idiopathic arthritis, polyarticular juvenile idiopathic arthritis, rheumatoid factor negative, oligoarticular juvenile idiopathic arthritis
Arthritis (HP:0001369)	*FAS*	Systemic juvenile idiopathic arthritis, polyarticular juvenile idiopathic arthritis, rheumatoid factor negative, oligoarticular juvenile idiopathic arthritis
**Immunodeficiencies affecting cellular and humoral immunity**		
Asthma (HP:0002099), Pneumonia (HP:0002090), Recurrent respiratory infections (HP:0002205)	*CARD11*	Respiratory system disease
Immunodeficiency (HP:0002721) Juvenile rheumatoid arthritis (HP:0005681), Polyarticular arthritis (HP:0005764)	*CD247*	Immune system disease Rheumatoid arthritis
Chronic hepatitis (HP:0200123) Enlarged tonsils (HP:0030812)	*CD40LG*	Chronic hepatitis B infection Tonsillectomy risk measurement
Neoplasm (HP:0002664)	*DOCK8*	Lung carcinoma
Autoimmunity (HP:0002960)	*ICOS*	Celiac disease Rheumatoid arthritis Type I diabetes mellitus Vitiligo Autoimmune thyroid disease, Hashimoto's thyroiditis, Graves disease
Eczema (HP:0000964) Hypothyroidism (HP:0000821) Pneumonia (HP:0002090) Autoimmunity (HP:0002960)	*IL7R*	Eczema Hypothyroidism Respiratory system disease Multiple sclerosis Type I diabetes mellitus Ulcerative colitis Ankylosing spondylitis
Attention deficit hyperactivity disorder (HP:0007018) Scoliosis (HP:0002650)	*LIG4*	Attention deficit hyperactivity disorder, oppositional defiant disorder measurementAdolescent idiopathic scoliosis
Eczema (HP:0000964)	*PTPRC*	Eczema
Recurrent sinopulmonary infections (HP:0005425)	*TFRC*	Respiratory system disease
**Predominantly antibody deficiencies**		
Neoplasm (HP:0002664) Chronic otitis media (HP:0000389); Recurrent otitis media (HP:0000403)	*TNFRSF13B*	Multiple myeloma Susceptibility to childhood ear infection measurement

* Also related to “Congenital defects of phagocyte number or function.”

**Figure 5:**
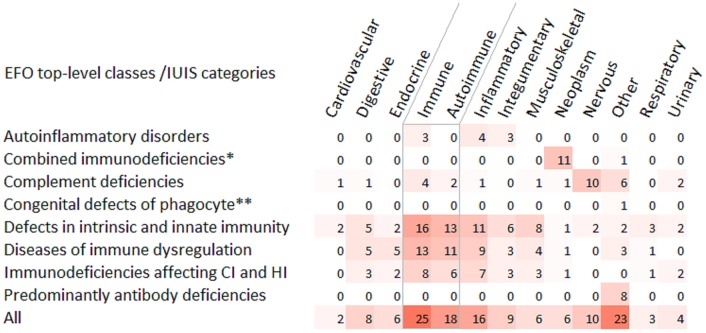
Heat-map matrix for the GWAS Catalog 2019 gene-set enrichment analysis. The IUIS genes were analyzed for their overrepresentation in common diseases/traits. The diseases/traits were classified according the Experimental Factor Ontology (EFO).The number of overrepresented common diseases/traits was indicated for EFO top-level categories (X-axis) within the IUIS categories of diseases (Y-axis). In the group of immune diseases, the subgroup of autoimmune conditions is highlighted with gray boundaries. ^*^Combined immunodeficiencies with associated or syndromic features; ^**^Congenital defects of phagocyte number or function. CI, cellular immunity; HI, humoral immunity.

Next, we performed the enrichment analysis with the GWAS Catalog 2019 gene set library within the Enrichr tool. The analysis was accomplished for eight IUIS categories and for the whole gene set (Figure 5, Supplementary Table 4). Disease classification was based on the Experimental Factor Ontology (EFO), the resource within the GWAS Catalog documentation. Two main results supporting previous findings are worthy of attention. First, enriched immune-related phenotypes were mainly represented by autoimmune conditions. Second, different combinations of ten genes (*RTEL1, TERT, TERC, STN1, ATM, SP110, SEMA3E, TBX1, DNMT3B, CDCA7*) related to the category “Combined immunodeficiencies with associated or syndromic features” were overrepresented in 11 neoplastic diseases/traits. “Other” mapped trait for this category was “telomere length.”

Findings presented in this section of our analysis are useful for understanding of phenotypic diversity of inborn errors of immunity. Gene sequencing techniques have become more widely available and will be soon routine methods. The analysis of genes whose genetic variants may influence clinical course of the genetic disease will be useful for management of patients with primary immunodeficiencies.

## Discussion

In this work, we provided some additional information on inborn errors of immunity summarized in the recent IUIS report (5). Using the ORPHANET resource we compared and characterized the IUIS and non-IUIS rare diseases associated with the IUIS genes. These genes were linked with a lot of phenotypes related to 29 top-level HPO categories in the HPO database. We estimated the phenotypic similarity between disease pairs associated with the same genes and between matched OMIM-ORPHANET disease pairs. Although the majority of inborn errors of immunity are considered monogenic, it is not always possible to identify damage in the corresponding gene. As far as medical and genomic data on rare diseases is progressively increasing, some of monogenic disorders may turn out to be associated with more than one gene. We reported additional relatively high score gene-disease associations for inborn errors of immunity. We found that all genes from the IUIS report were associated with several or many complex diseases. Genetic overlap between rare and common diseases is an important and clinically relevant problem, which is currently at the forefront of research (17, 18). We showed the enrichment of gene sets related to several IUIS categories with neoplastic and autoimmune diseases from the GWAS Catalog and reported individual genes with phenotypic overlap between inborn errors of immunity and GWAS diseases/traits. Our work was aimed not only at gaining new knowledge, but also at demonstrating the possibilities and the necessity of communication between clinicians and geneticists to ensure progress in the direction of precision medicine of primary immunodeficiencies. We conclude with highlighting several important aspects contributing to the overview of phenotypic diversity of inborn errors of immunity.

Linking genes of inborn errors of immunity with the HPO terms showed a huge variety of symptoms with the involvement of many organs and systems. With the use of a phenotype-based tool Gene ORGANizer, we demonstrated that gene sets related to HPO categories were most often enriched in immune, lymphatic, and corresponding body systems (cardiovascular genes in cardiovascular system, endocrine genes in endocrine system, etc.); however no enrichment was revealed for nervous system. The latter fact may be due to the nervous system related content of background information in Gene ORGANizer, which is discussed in the relevant paper (13). Besides, we assumed that certain nervous system abnormalities may be secondary relative abnormalities in other body systems. Neurological complications may occur as a result of infections (19), vascular problems (20), metabolic disturbances (21), treatment complications (22) and other health problems in inborn errors of immunity. The diagnostic and therapeutic delay in PIDs may lead to the development of irreversible organ damage (23–25). The diversity of symptoms complicates their diagnosis as features accompanying primary immunodeficiencies (26). The correct and quick diagnosis of a rare disease strongly depends on the associated clinical phenotypes and their recognition by physicians (1). We believe that our report on frequent and very frequent symptoms in inborn errors of immunity may be useful for accelerated and effective management of patients with primary immunodeficiencies.

An interesting finding in our work stems from the analysis of the phenotypic similarity between gene-related phenotypes. The Jaccard/Tanimoto coefficients for the HPO terms related to different rare diseases associated with the same gene were low, pointing to high pleiotropy. These results correlate with high genetic pleiotropy in diseases and traits from DisGeNET, however, pleiotropy due to rare alleles might be more pronounced (27). In contrast to the low Jaccard/Tanimoto coefficients for the HPO terms, the coefficients for the top-level HPO categories were relatively high. This observation supports a modular view of pleiotropy (28). A modular character of gene–trait relationships in particular means that mutations in a certain gene may lead to the development of different, partially overlapping combinations of clinical signs and symptoms with non-random clustering (29). Diseases associated with the same gene might share common biological pathways and affect the same anatomical systems.

Our study reported 25 inborn errors of immunity associated with more than one gene. These inborn errors of immunity were associated with two or more unrelated genes, gene clusters or unrelated genes along with gene clusters (20 gene clusters of 2 to 16 genes). Gene clusters were retrieved from protein-protein interaction (PPI) information, which is increasingly used for the identification of new disease-related proteins since the disease-related genes tend to form closely interconnected clusters in the protein network (30, 31). Some “monogenic” diseases (e.g., Aicardi-Goutieres syndrome 2 (AGS2), Bare lymphocyte syndrome, type II, complementation group A/C/D/E) were associated with one top gene and several else genes more closely linked to another disorder from a given phenotypic spectrum. According DisGeNET, genes *TREX1, ADAR, IFIH1*, and *SAMHD1* associated with AGS1, AGS6, AGS7, and AGS5, respectively, may be also implicated in AGS2. These genes are interconnected within a biological network including also *USP18* and the top associated gene *RNASEH2B*.

Investigations of phenotypic and genetic overlap between monogenic and common diseases are focused on expanding knowledge on both common and rare diseases. Genes associated with rare diseases are usually functionally related to disease-relevant pathways. It has been recently hypothesized that phenotypic overlap might be a general trend for rare and common diseases associated with the same gene (17). Phenotypic overlap between rare and common diseases linked by genomic location indirectly supports the common variant association with the common disease. Genes with common variants associated with a common trait might also carry large-effect variants at the extremes of the trait (32). For most traits, an inverse relationship between the effect size and the allele frequency has been shown (33), however, many small-effect genetic variants in sum may play a greater role than rare large-effect mutations (34). Given these observations, we speculate that common variants in relevant genes and relevant metabolic pathways might modify monogenic diseases in terms of their clinical course and response to treatment. The influence of genetic background on the “monogenic” disease phenotype has been shown for several diseases (35). We reported here that common variants in three genes with gene function related to telomere maintenance (*RTEL1*, Regulator of Telomere Elongation Helicase 1; *TERC*, Telomerase RNA Component; *TERT*, Telomerase Reverse Transcriptase) have been associated with telomere length and multiple neoplasms in GWAS. Both HPO terms and ORPHANET classifications for rare diseases associated with these genes include neoplasms. The second example from our study is related to autoimmune conditions. Four genes *CTLA4, IL2RA, ICOS*, and *IL7R* have been associated with a group of autoimmune diseases in GWAS and with autoimmunity in the HPO database. We assume that mutations, rare large-effect variants and polymorphisms influencing the same pathogenic processes might cooperate in determining the severity of the rare disease phenotype.

The main findings of the study and their possible implications are as follows: (i) Genes associated with inborn errors of immunity are pleiotropic being involved in a broad, however non-random spectrum of phenotypic abnormalities. (ii) Summary of frequent and very frequent clinical signs and symptoms may be useful for diagnosis in non-obvious cases and for early detection of possible complications. (iii) Some, if not many inborn errors of immunity can actually be associated with more than one gene. If genetic damage is unknown, the search for causal genes can first start among those encoding interacting proteins. (iv) Phenotypic diversity of inborn errors of immunity depends not only on time of diagnosis and treatment characteristics, but also on mutation penetrance and expressivity, the latter being linked, in particular, to genetic background. Common genetic variants in certain genes might contribute to even greater risk of the development of autoimmune and neoplastic diseases in inborn errors of immunity.

In summary, based on the analysis of gene-disease data from the recent IUIS report (5) we presented some additional knowledge on the relevant genes and phenotypes of inborn errors of immunity. The rapid development of genomic technologies is making possible promotion of personalized medicine. We exemplified the possibilities of using of genomic technologies in the field of inborn errors of immunity. This information and instances may be useful for clinical differential diagnostics and for the development of translational and basic research of primary immunodeficiencies.

## Data Availability Statement

The datasets for this manuscript are available in Supplementary Materials or upon request.

## Ethics Statement

Ethical review and approval was not required for the study on human participants in accordance with the local legislation and institutional requirements. Written informed consent for participation was not required for this study in accordance with the national legislation and the institutional requirements.

## Author Contributions

LS designed the study. EC and LA collected data and prepared figures. LS and SL analyzed and interpreted data. LS wrote the manuscript. LS, EC, LA, and SL approved the final version.

### Conflict of Interest

The authors declare that the research was conducted in the absence of any commercial or financial relationships that could be construed as a potential conflict of interest.
