# Supplementary material for: Gene- and Disease-Based Expansion of the Knowledge on Inborn Errors of Immunity
Source: Front Immunol. 2019 Oct 21;10:2475. doi: 10.3389/fimmu.2019.02475 (PMC6816315; doi:10.3389/fimmu.2019.02475)
Supplement: Supplementary file 2 [file Table_2.DOCX]

Supplementary Table 2. The list of 110 diseases with available data for the abundance of frequent and very frequent clinical signs and symptoms.

| No. | Disease |
| --- | --- |
| 1 | 22q11.2 deletion syndrome |
| 2 | 3-methylglutaconic aciduria type 7 |
| 3 | Aicardi-Goutières syndrome |
| 4 | Albers-Schönberg osteopetrosis |
| 5 | Alymphoid cystic thymic dysgenesis |
| 6 | Ataxia-pancytopenia syndrome |
| 7 | Ataxia-telangiectasia |
| 8 | Atypical hemolytic-uremic syndrome with I factor anomaly |
| 9 | Autoimmune enteropathy and endocrinopathy-susceptibility to chronic infections syndrome |
| 10 | Autoimmune hemolytic anemia-autoimmune thrombocytopenia-primary immunodeficiency syndrome |
| 11 | Autoimmune lymphoproliferative syndrome |
| 12 | Autoimmune polyendocrinopathy type 1 |
| 13 | Autosomal agammaglobulinemia |
| 14 | Autosomal dominant hyper-IgE syndrome |
| 15 | Autosomal dominant severe congenital neutropenia |
| 16 | Autosomal recessive malignant osteopetrosis |
| 17 | Baraitser-Winter cerebrofrontofacial syndrome |
| 18 | Barth syndrome |
| 19 | Blau syndrome |
| 20 | Bloom syndrome |
| 21 | Cartilage-hair hypoplasia |
| 22 | Cernunnos-XLF deficiency |
| 23 | CHARGE syndrome |
| 24 | Chédiak-Higashi syndrome |
| 25 | Cherubism |
| 26 | Chronic granulomatous disease |
| 27 | Chronic mucocutaneous candidiasis |
| 28 | CINCA syndrome |
| 29 | Cohen syndrome |
| 30 | Combined immunodeficiency due to DOCK8 deficiency |
| 31 | Combined immunodeficiency due to partial RAG1 deficiency |
| 32 | Combined immunodeficiency due to ZAP70 deficiency |
| 33 | Combined immunodeficiency-enteropathy spectrum |
| 34 | Common variable immunodeficiency |
| 35 | Cyclic neutropenia |
| 36 | Cystic fibrosis |
| 37 | Deafness-lymphedema-leukemia syndrome |
| 38 | Deficiency in anterior pituitary function-variable immunodeficiency syndrome |
| 39 | Developmental malformations-deafness-dystonia syndrome |
| 40 | Dyskeratosis congenita |
| 41 | Epidermodysplasia verruciformis |
| 42 | Familial acute necrotizing encephalopathy |
| 43 | Familial cold urticaria |
| 44 | Familial infantile bilateral striatal necrosis |
| 45 | Familial Mediterranean fever |
| 46 | Familial melanoma |
| 47 | Griscelli syndrome type 12 |
| 48 | Griscelli syndrome type 13 |
| 49 | Griscelli syndrome type 3 |
| 50 | Griscelli syndrome type 4 |
| 51 | Griscelli syndrome type 5 |
| 52 | Griscelli syndrome type 6 |
| 53 | Griscelli syndrome type 8 |
| 54 | Griscelli syndrome type 9 |
| 55 | H syndrome |
| 56 | Hennekam syndrome |
| 57 | Hepatic veno-occlusive disease-immunodeficiency syndrome |
| 58 | Hereditary angioedema type 1 |
| 59 | Hereditary chronic pancreatitis |
| 60 | Hereditary folate malabsorption |
| 61 | Hereditary pulmonary alveolar proteinosis |
| 62 | Hoyeraal-Hreidarsson syndrome |
| 63 | Hyperimmunoglobulinemia D with periodic fever |
| 64 | ICF syndrome |
| 65 | Idiopathic aplastic anemia |
| 66 | Idiopathic bronchiectasis |
| 67 | Idiopathic pulmonary fibrosis |
| 68 | Immune dysregulation-polyendocrinopathy-enteropathy-X-linked syndrome |
| 69 | Immunodeficiency by defective expression of HLA class 2 |
| 70 | Immunodeficiency due to interleukin-1 receptor-associated kinase-4 deficiency |
| 71 | Kabuki syndrome |
| 72 | LIG4 syndrome |
| 73 | Lynch syndrome |
| 74 | Majeed syndrome |
| 75 | Mendelian susceptibility to mycobacterial diseases due to partial IRF8 deficiency |
| 76 | Mevalonic aciduria |
| 77 | Microcephalic osteodysplastic primordial dwarfism types I and III |
| 78 | MOGS-CDG |
| 79 | Muckle-Wells syndrome |
| 80 | Multiple intestinal atresia |
| 81 | Netherton syndrome |
| 82 | Neutrophil immunodeficiency syndrome |
| 83 | Nijmegen breakage syndrome |
| 84 | Omenn syndrome |
| 85 | Papillon-Lefèvre syndrome |
| 86 | Periodontal Ehlers-Danlos syndrome |
| 87 | Pityriasis rubra pilaris |
| 88 | Primary immunodeficiency syndrome due to p14 deficiency |
| 89 | Prolidase deficiency |
| 90 | Pyogenic arthritis-pyoderma gangrenosum-acne syndrome |
| 91 | Pyogenic bacterial infections due to MyD88 deficiency |
| 92 | Reticular dysgenesis |
| 93 | RIDDLE syndrome |
| 94 | Schimke immuno-osseous dysplasia |
| 95 | Severe combined immunodeficiency due to adenosine deaminase deficiency |
| 96 | Severe combined immunodeficiency due to DCLRE1C deficiency |
| 97 | Shwachman-Diamond syndrome |
| 98 | Spondyloenchondrodysplasia |
| 99 | T-B+ severe combined immunodeficiency due to gamma chain deficiency |
| 100 | T-B+ severe combined immunodeficiency due to JAK3 deficiency |
| 101 | Tetralogy of Fallot |
| 102 | Transcobalamin deficiency |
| 103 | Tubular aggregate myopathy |
| 104 | Tumor necrosis factor receptor 1 associated periodic syndrome |
| 105 | Vici syndrome |
| 106 | WHIM syndrome |
| 107 | Wiskott-Aldrich syndrome |
| 108 | X-linked agammaglobulinemia |
| 109 | X-linked lymphoproliferative disease |
| 110 | X-linked severe congenital neutropenia |
