# Supplementary material for: Gene- and Disease-Based Expansion of the Knowledge on Inborn Errors of Immunity
Source: Front Immunol. 2019 Oct 21;10:2475. doi: 10.3389/fimmu.2019.02475 (PMC6816315; doi:10.3389/fimmu.2019.02475)
Supplement: Supplementary file 3 [file Table_3.DOCX]

Supplementary Table 3. IUIS/OMIM diseases with gene-disease association scores according the DisGeNET database

| No. | Disease | S_1_ (≥ 0.3) | | S_2_  _#_ | S_3_  _#_ |
| --- | --- | --- | --- | --- | --- |
|  |  | # | Gene symbols (Score) |  |  |
| 1 | Acne inversa, familial, 3 | 0 | - | 1 | 0 |
| 2 | Agammaglobulinemia 7, autosomal recessive | 1 | *PIK3R1*(0.4) | 0 | 0 |
| 3 | Agammaglobulinemia 8, autosomal dominant | 0 | *-* | 1 | 0 |
| 4 | Aicardi-Goutieres syndrome 2 | 6 | *RNASEH2B* (0.6), *TREX1* (0.5), *ADAR* (0.3), *IFIH1* (0.3), *USP18* (0.3), *SAMHD1* (0.3) | 0 | 0 |
| 5 | Aicardi-Goutieres syndrome 3 | 1 | *RNASEH2C* (0.6) | 1 | 0 |
| 6 | Aicardi-Goutieres syndrome 4 | 1 | *RNASEH2A* (0.6) | 1 | 0 |
| 7 | Aicardi-Goutieres syndrome 6 | 1 | *ADAR* (0.4) | 1 | 0 |
| 8 | Aicardi-Goutieres syndrome 7 | 1 | *IFIH1* (0.4) | 1 | 0 |
| 9 | Alpha/beta t-cell lymphopenia with gamma/delta t-cell expansion, severe cytomegalovirus infection, and autoimmunity | 1 | *RAG1* (0.7) | 0 | 0 |
| 10 | Aplastic anemia | 7 | *TERT* (0.69), *IFNG* (0.6), *NBN* (0.42), *PRF1* (0.4), *CSF3* (0.37), *CSF2* (0.34), *SBDS* (0.32) | 3 | 138 |
| 11 | Autoimmune disease, multisystem, infantile-onset, 1 | 1 | *STAT3* (0.7) | 0 | 0 |
| 12 | Autoimmune disease, multisystem, with facial dysmorphism | 1 | *ITCH* (0.6) | 0 | 0 |
| 13 | Autoimmune interstitial lung, joint, and kidney disease | 1 | *COPA* (0.71) | 0 | 0 |
| 14 | Autoimmune lymphoproliferative syndrome | 6 | *FAS* (1.0), *FASLG* (0.93), *CASP10* (0.34), *NRAS* (0.32), *PRKCD* (0.31), *CASP8* (0.3) | 0 | 24 |
| 15 | Autoimmune lymphoproliferative syndrome, type IB | 2 | *FASLG* (0.4), *FAS* (0.3) | 0 | 1 |
| 16 | Autoimmune lymphoproliferative syndrome, type III | 1 | *PRKCD* (0.4) | 0 | 0 |
| 17 | Autoinflammation with arthritis and dyskeratosis | 1 | *NLRP1* (0.6) | 0 | 0 |
| 18 | Autoinflammation with infantile enterocolitis | 2 | *NLRC4* (0.7) | 0 | 1 |
| 19 | Autoinflammation, panniculitis, and dermatosis syndrome | 1 | *OTULIN* (0.7) | 0 | 0 |
| 20 | Autoinflammatory syndrome, familial, Behcet-like | 1 | *TNFAIP3* (0.7) | 0 | 0 |
| 21 | Bare lymphocyte syndrome, type I | 4 | *TAP2* (0.51), *TAPBP* (0.51), *TAP1* (0.5), *B2M* (0.3) | 0 | 4 |
| 22 | Bare lymphocyte syndrome, type II, complementation group A | 3 | *CIITA* (0.4), *RFX5* (0.3), *RFXANK* (0.3) | 0 | 0 |
| 23 | Bare lymphocyte syndrome, type II, complementation group C | 3 | *CIITA* (0.3), *RFX5* (0.3), *RFXANK* (0.3) | 0 | 0 |
| 24 | Bare lymphocyte syndrome, type II, complementation group D | 3 | *CIITA* (0.3), *RFX5* (0.3), *RFXANK* (0.3) | 1 | 0 |
| 25 | Bare lymphocyte syndrome, type II, complementation group E | 3 | *RFX5* (0.4),  *CIITA* (0.3), *RFXANK* (0.3) | 0 | 0 |
| 26 | Bloom syndrome | 2 | *BLM* (1.0), *UNG* (0.35) | 1 | 76 |
| 27 | Bone marrow failure syndrome 2 | 4 | *ERCC6L2* (0.6), *CSF2* (0.3), *SRP72* (0.3), *DNAJC21* (0.3) | 0 | 0 |
| 28 | C1q deficiency (C1q deficiency due to defects in C1QA, C1q deficiency due to defects in C1QB, C1q deficiency due to defects in C1QC) | 3 | *C1QB* (0.71), *C1QC* (0.6), *C1QA* (0.4) | 0 | 2 |
| 29 | C3 deficiency | 0 | *-* | 0 | 2 |
| 30 | C9 deficiency | 1 | *C9* (0.65) | 0 | 0 |
| 31 | Candidiasis, familial, 8 | 1 | *TRAF3IP2* (0.6) | 1 | 0 |
| 32 | Candidiasis, familial, 9 | 1 | *IL17RC* (0.4) | 0 | 0 |
| 33 | Cartilage-hair hypoplasia | 1 | *RMRP* (0.6) | 0 | 26 |
| 34 | CD8 deficiency, familial | 1 | *CD8A* (0.7) | 1 | 0 |
| 35 | Cerebroretinal microangiopathy with calcifications and cysts 2 | 1 | *STN1* (0.4) | 0 | 0 |
| 36 | CHARGE syndrome | 2 | *CHD7* (1.0), *SEMA3E* (0.6) | 2 | 58 |
| 37 | Chediak-Higashi syndrome | 1 | *LYST* (1.0) | 0 | 19 |
| 38 | Cherubism | 1 | *SH3BP2* (1.0) | 0 | 0 |
| 39 | Chilblain lupus 2 | 1 | *SAMHD1* (0.5) | 0 | 0 |
| 40 | Cohen syndrome | 1 | *VPS13B* (0.8) | 0 | 5 |
| 41 | Colorectal cancer, hereditary nonpolyposis, type 4 | 1 | *PMS2* (0.7) | 0 | 0 |
| 42 | Colorectal cancer, hereditary nonpolyposis, type 5 | 1 | *MSH6* (0.7) | 0 | 0 |
| 43 | Combined cellular and humoral immune defects with granulomas (RAG1 deficiency; RAG2 deficiency) | 2 | *RAG1* (0.7), *RAG2* (0.7) | 0 | 0 |
| 44 | Complement component 4, partial deficiency of | 1 | *SERPING1* (0.5) | 0 | 0 |
| 45 | Complement factor B deficiency | 1 | *CFB* (0.4) | 0 | 0 |
| 46 | Complement factor D deficiency | 1 | *CFD* (0.7) | 0 | 0 |
| 47 | Complement Factor H Deficiency | 1 | *CFH* (0.79) | 0 | 3 |
| 48 | Congenital disorder of glycosylation, type IIb | 1 | *MOGS* (0.72) | 1 | 0 |
| 49 | Cystic fibrosis | 9 | *CFTR* (1.0), *SCNN1B* (0.6), *TGFB1* (0.6), *DCTN4* (0.52), *SCNN1A* (0.37), *SCNN1G* (0.36), *CLCA4* (0.34), *STX1A* (0.31), *TNFRSF1A* (0.31) | 17 | 567 |
| 50 | DiGeorge syndrome | 16 | *TBX1* (0.9), *COMT* (0.6), *CRKL* (0.51), *FGF8* (0.5), *HIRA* (0.4), *DGCR* (0.4), *UFD1* (0.33), *DGCR6* (0.33), *DGCR2* (0.32), *DGCR8* (0.31), *GP1BB* (0.31), *ARVCF* (0.3), *JMJD1C* (0.3), *SEC24C* (0.3), *ESS2* (0.3), *RREB1* (0.3) | 12 | 45 |
| 51 | Dursun syndrome | 1 | *G6PC3* (0.4) | 1 | 0 |
| 52 | Dyskeratosis congenita, autosomal dominant 2 | 1 | *TERT* (0.6) | 1 | 1 |
| 53 | Dyskeratosis congenita, autosomal dominant 4 | 1 | *RTEL1* (0.6) | 1 | 0 |
| 54 | Dyskeratosis congenita, autosomal recessive 1 | 1 | *NOP10* (0.4) | 2 | 0 |
| 55 | Dyskeratosis congenita, autosomal recessive 2 | 1 | *NHP2* (0.6) | 0 | 0 |
| 56 | Dyskeratosis congenita, autosomal recessive 5 | 1 | *RTEL1* (0.6) | 1 | 0 |
| 57 | Dyskeratosis congenita, autosomal recessive 6 | 1 | *PARN* (0.6) | 1 | 0 |
| 58 | Ehlers-Danlos syndrome, periodontal type, 2 | 1 | C1S (0.6) | 0 | 0 |
| 59 | Emberger syndrome | 1 | GATA2 (0.75) | 0 | 1 |
| 60 | Epidermodysplasia verruciformis (EVER1 deficiency; EVER2 deficiency) | 2 | *TMC8* (0.7), *TMC6* (0.7) | 0 | 19 |
| 61 | Familial cold autoinflammatory syndrome 2 | 1 | *NLRP12* (0.6) | 0 | 0 |
| 62 | Familial cold autoinflammatory syndrome 3 | 1 | *PLCG2* (0.3) | 0 | 0 |
| 63 | Familial cold autoinflammatory syndrome 4 | 1 | *NLRC4* (0.6) | 0 | 0 |
| 64 | Folate malabsorption, hereditary | 1 | *SLC46A1* (0.8) | 0 | 1 |
| 65 | Granulomatous disease, chronic, autosomal recessive, cytochrome B-positive, type III | 1 | *NCF4* (0.6) | 1 | 0 |
| 66 | Griscelli syndrome, type 2 | 1 | *RAB27A* (0.8) | 0 | 2 |
| 67 | Haim-Munk syndrome | 1 | *CTSC* (0.75) | 0 | 0 |
| 68 | Hemolytic uremic syndrome, atypical, susceptibility to, 1 | 1 | *CFH* (0.4) | 2 | 0 |
| 69 | Hemolytic uremic syndrome, atypical, susceptibility to, 2 | 1 | *CD46* (0.6) | 0 | 0 |
| 70 | Hemolytic uremic syndrome, atypical, susceptibility to, 3 | 1 | *CFI* (0.6) | 0 | 0 |
| 71 | Hemolytic uremic syndrome, atypical, susceptibility to, 4 | 1 | *CFB* (0.6) | 0 | 0 |
| 72 | Hemolytic uremic syndrome, atypical, susceptibility to, 5 | 1 | *C3* (0.6) | 0 | 0 |
| 73 | Hemolytic uremic syndrome, atypical, susceptibility to, 6 | 1 | *THBD* (0.6) | 0 | 0 |
| 74 | Hemophagocytic lymphohistiocytosis, familial, 2 | 1 | *PRF1* (0.85) | 0 | 3 |
| 75 | Hemophagocytic lymphohistiocytosis, familial, 3 | 1 | *UNC13D* (0.64) | 0 | 3 |
| 76 | Hemophagocytic lymphohistiocytosis, familial, 4 | 1 | *STX11* (0.63) | 0 | 0 |
| 77 | Hemophagocytic lymphohistiocytosis, familial, 5 | 1 | *STXBP2* (0.6) | 0 | 0 |
| 78 | Hennekam lymphangiectasia-lymphedema syndrome 1 | 1 | *CCBE1* (0.3) | 0 | 0 |
| 79 | Hennekam lymphangiectasia-lymphedema syndrome 2 | 1 | *FAT4* (0.4) | 0 | 0 |
| 80 | Hepatic venoocclusive disease with immunodeficiency | 1 | *SP110* (0.61) | 1 | 0 |
| 81 | Hermansky-Pudlak syndrome 2 | 1 | *AP3B1* (0.94) | 1 | 3 |
| 82 | Immunodeficiency 12 | 1 | *MALT1* (0.7) | 0 | 0 |
| 83 | Immunodeficiency 16 | 1 | *TNFRSF4* (0.71) | 0 | 1 |
| 84 | Immunodeficiency 18, SCID variant | 1 | *CD3E* (0.5) | 0 | 0 |
| 85 | Immunodeficiency 19 | 1 | *CD3D* (0.41) | 0 | 0 |
| 86 | Immunodeficiency 20 | 1 | *FCGR3A* (0.6) | 0 | 0 |
| 87 | Immunodeficiency 22 | 1 | *LCK* (0.7) | 0 | 0 |
| 88 | Immunodeficiency 23 | 1 | PGM3 (0.7) | 0 | 0 |
| 89 | Immunodeficiency 24 | 1 | *CTPS1* (0.6) | 0 | 0 |
| 90 | Immunodeficiency 30 | 1 | *IL12RB1* (0.72) | 0 | 0 |
| 91 | Immunodeficiency 36 | 1 | *PIK3R1* (0.4) | 0 | 0 |
| 92 | Immunodeficiency 40 | 1 | *DOCK2* (0.7) | 0 | 0 |
| 93 | Immunodeficiency 42 | 1 | *RORC* (0.7) | 0 | 0 |
| 94 | Immunodeficiency 44 | 2 | *STAT2* (0.6), *IFNAR2* (0.3) | 0 | 0 |
| 95 | Immunodeficiency 45 | 1 | *IFNAR2* (0.6), *STAT2* (0.3) | 0 | 0 |
| 96 | Immunodeficiency 46 | 1 | *TFRC* (0.7) | 0 | 0 |
| 97 | Immunodeficiency 50 | 1 | *MSN* (0.7) | 0 | 0 |
| 98 | Immunodeficiency 51 | 1 | *IL17RA* (0.3) | 0 | 0 |
| 99 | Immunodeficiency 52 | 1 | *LAT* (0.4) | 0 | 0 |
| 100 | Immunodeficiency 8 | 1 | *CORO1A* (0.7) | 0 | 0 |
| 101 | Immunodeficiency due to defect in MAPBP-interacting protein | 1 | *LAMTOR2* (0.5) | 0 | 0 |
| 102 | Immunodeficiency, common variable, 1 | 0 | *-* | 1 | 0 |
| 103 | Immunodeficiency, common variable, 10 | 1 | *NFKB2* (0.41) | 0 | 1 |
| 104 | Immunodeficiency, common variable, 11 | 1 | *IL21* (0.6) | 1 | 0 |
| 105 | Immunodeficiency, common variable, 12 | 0 | *-* | 1 | 0 |
| 106 | Immunodeficiency, common variable, 13 | 1 | *IKZF1* (0.6) | 0 | 0 |
| 107 | Immunodeficiency, common variable, 2 | 1 | *TNFRSF13B* (0.4) | 0 | 0 |
| 108 | Immunodeficiency, common variable, 6 | 0 | *-* | 1 | 0 |
| 109 | Immunodeficiency, common variable, 7 | 0 | *-* | 1 | 0 |
| 110 | Immunodeficiency, common variable, 8, with autoimmunity | 1 | *LRBA* (0.7) | 0 | 0 |
| 111 | Immunodeficiency-centromeric instability-facial anomalies syndrome 2 | 1 | *ZBTB24* (0.61) | 0 | 0 |
| 112 | Immunodeficiency-centromeric instability-facial anomalies syndrome 3 | 1 | *CDCA7* (0.6) | 0 | 0 |
| 113 | Iimmunodeficiency-centromeric instability-facial anomalies syndrome 4 | 1 | *HELLS* (0.6) | 0 | 0 |
| 114 | Immunoskeletal dysplasia with neurodevelopmental abnormalities | 1 | *EXTL3* (0.6) | 0 | 0 |
| 115 | Infantile liver failure syndrome 2 | 1 | *NBAS* (0.7) | 0 | 0 |
| 116 | Infections, recurrent, with encephalopathy, hepatic dysfunction, and cardiovascular malformations | 1 | *FADD* (0.6) | 0 | 0 |
| 117 | Inflammatory skin and bowel disease, neonatal, 1 | 1 | *ADAM17* (0.4) | 0 | 0 |
| 118 | Interleukin 1 receptor antagonist deficiency | 1 | *IL1RN* (0.64) | 0 | 0 |
| 119 | Immunoskeletal dysplasia with neurodevelopmental abnormalities | 1 | *EXTL3* (0.6) | 0 | 2 |
| 120 | IRAK4 deficiency | 1 | *IRAK4* (0.71) | 0 | 3 |
| 121 | Kabuki syndrome 2 | 0 | - | 1 | 0 |
| 122 | Leukemia, acute myeloid, susceptibility to | 4 | *SETD2* (0.3), *CEBPA* (0.3), *SETBP1* (0.3), *JAK2* (0.3) | 1 | 0 |
| 123 | Leukocyte adhesion deficiency, type III | 1 | *FERMT3* (0.92) | 1 | 5 |
| 124 | LIG4 syndrome | 2 | *LIG4* (0.99), *XRCC4* (0.3) | 0 | 0 |
| 125 | Lymphoproliferative syndrome 1 | 1 | *ITK* (0.4) | 0 | 0 |
| 126 | Lymphoproliferative syndrome 2 | 1 | *CD27* (0.61) | 1 | 0 |
| 127 | Lymphoproliferative syndrome, X-Linked, 2 | 1 | *XIAP* (0.46) | 0 | 7 |
| 128 | Majeed syndrome | 1 | *LPIN2* (0.74) | 0 | 7 |
| 129 | MASP2 deficiency | 1 | *MASP2* (0.6) | 0 | 3 |
| 130 | Mevalonic aciduria | 1 | *MVK* (0.8) | 0 | 3 |
| 131 | Microcephalic osteodysplastic primordial dwarfism, type i | 1 | *RNU4ATAC* (0.37) | 1 | 1 |
| 132 | Mirage syndrome | 1 | *SAMD9* (0.6) | 0 | 0 |
| 133 | Muckle-Wells syndrome | 4 | *NLRP3* (0.8), *NLRC4* (0.3), *MME* (0.3), *PLCG2* (0.3) | 0 | 24 |
| 134 | Myelokathexis, isolated | 0 | *-* | 1 | 0 |
| 135 | Myopathy, tubular aggregate, 1 | 2 | *CASQ1* (0.3), STIM1 (0.3) | 0 | 0 |
| 136 | Myopathy, tubular aggregate, 2 | 1 | *ORAI1* (0.4) | 0 | 0 |
| 137 | Neutropenia, severe congenital, 5, autosomal recessive | 1 | *VPS45* (0.7) | 1 | 0 |
| 138 | Neutropenia, severe congenital, 6, autosomal recessive | 1 | *JAGN1* (0.7) | 1 | 0 |
| 139 | Neutropenia, severe congenital, 7, autosomal recessive | 1 | *CSF3R* (0.7) | 0 | 0 |
| 140 | Neutropenia, severe congenital, X-linked | 1 | *WAS* (0.7) | 1 | 1 |
| 141 | Neutrophil immunodeficiency syndrome | 1 | *RAC2* (0.71) | 0 | 0 |
| 142 | Nijmegen breakage syndrome | 1 | *NBN* (0.9) | 4 | 56 |
| 143 | Omenn syndrome | 15 | *RAG2* (0.8), *RAG1* (0.6), *DCLRE1C* (0.55), *IL7R* (0.33), *IL2RG* (0.32), *CHD7* (0.31), *CD3E* (0.31), *ZAP70* (0.31), *ADA* (0.31), *CD3D* (0.3), *TFRC* (0.3), *AK2* (0.3), *RMRP* (0.3), *LIG4* (0.3), *JAK3* (0.3) | 0 | 16 |
| 144 | Osteopetrosis, autosomal recessive 1 | 1 | *TCIRG1* (0.8) | 0 | 0 |
| 145 | Osteopetrosis, autosomal recessive 4 | 1 | *CLCN7* (0.8) | 0 | 0 |
| 146 | Osteopetrosis, autosomal recessive 5 | 1 | *OSTM1* (0.6) | 0 | 0 |
| 147 | Osteopetrosis, autosomal recessive 6 | 3 | *PLEKHM1* (0.8), *CLCN7* (0.3), *TCIRG1* (0.3) | 0 | 0 |
| 148 | Osteopetrosis, autosomal recessive 7 | 1 | *TNFRSF11A* (0.7) | 0 | 0 |
| 149 | Osteopetrosis, autosomal recessive 8 | 1 | *SNX10* (0.6) | 0 | 0 |
| 150 | Pityriasis rubra pilaris | 1 | *CARD14* (0.76) | 0 | 9 |
| 151 | Poikiloderma with neutropenia | 1 | *USB1* (0.7) | 0 | 12 |
| 152 | Polyglucosan body myopathy 1 with or without immunodeficiency | 1 | *RBCK1* (0.4) | 0 | 0 |
| 153 | Properdin deficiency, X-linked | 1 | *CFP* (0.7) | 0 | 0 |
| 154 | Pseudo-Torch syndrome 2 | 1 | *USP18* (0.4) | 0 | 0 |
| 155 | Psoriasis 15, pustular, susceptibility to | 1 | *AP1S3* (0.4) | 0 | 0 |
| 156 | Psoriasis 2 | 1 | *CARD14* (0.41) | 0 | 0 |
| 157 | Pulmonary fibrosis and/or bone marrow failure, telomere-related, 1 | 1 | *TERT* (0.6) | 0 | 0 |
| 158 | Pulmonary fibrosis and/or bone marrow failure, telomere-related, 4 | 1 | *PARN* (0.6) | 0 | 0 |
| 159 | Reticular dysgenesis | 1 | *AK2* (0.74) | 0 | 0 |
| 160 | Riddle Syndrome | 1 | *RNF168* (0.83) | 0 | 2 |
| 161 | Roifman syndrome | 1 | *RNU4ATAC* (0.51) | 0 | 1 |
| 162 | Schimke immunoosseous dysplasia | 1 | *SMARCAL1* (1.0) | 0 | 6 |
| 163 | Severe combined immunodeficiency with microcephaly, growth retardation, and sensitivity to ionizing radiation | 1 | *NHEJ1* (0.71) | 0 | 1 |
| 164 | Severe combined immunodeficiency, B cell-negative | 0 | *-* | 2 | 0 |
| 165 | Siideroblastic anemia with B-cell immunodeficiency, periodic fevers, and developmental delay | 1 | *TRNT1* (0.62) | 0 | 0 |
| 166 | Singleton-Merten syndrome 1 | 1 | *IFIH1* (0.4) | 0 | 0 |
| 167 | Singleton-Merten syndrome 2 | 1 | *DDX58* (0.4) | 0 | 0 |
| 168 | Specific granule deficiency | 2 | *CEBPE* (0.61), *SMARCD2* (0.6) | 0 | 6 |
| 169 | Specific granule deficiency 2 | 2 | *SMARCD2* (0.4), *CEBPE* (0.3) | 0 | 0 |
| 170 | Spondyloenchondrodysplasia with immune dysregulation | 1 | *ACP5* (0.6) | 1 | 0 |
| 171 | STING-associated vasculopathy, infantile-onset | 1 | *TMEM173* (0.72) | 0 | 0 |
| 172 | Surfactant metabolism dysfunction, pulmonary, 4 | 1 | *CSF2RA* (0.4) | 0 | 1 |
| 173 | Surfactant metabolism dysfunction, pulmonary, 5 | 1 | *CSF2RB* (0.4) | 0 | 0 |
| 174 | Tetralogy of fallot | 16 | *ZFPM2* (0.98), *NKX2-5* (0.96), *GATA4* (0.8), *JAG1* (0.8), *GATA6* (0.73), *GDF1* (0.71), *GJA5* (0.62), *CITED2* (0.6), *TBX1* (0.46), *GATA5* (0.42), *NKX2-6* (0.4), *FOXC2* (0.4), *HAND2* (0.31), *FOXC1* (0.3), *FLT4* (0.3), *FOXH1* (0.3) | 62 | 46 |
| 175 | Thrombocytopenia, X-linked, intermittent | 1 | *WAS* (0.6) | 0 | 0 |
| 176 | Transcobalamin II deficiency | 1 | *TCN2* (0.63) | 0 | 0 |
| 177 | Van Maldergem syndrome 2 | 1 | *FAT4* (0.4) | 0 | 0 |
| 178 | WHIM syndrome | 1 | *CXCR4* (0.9) | 0 | 7 |
| 179 | Wiskott-Aldrich syndrome | 2 | *WAS* (1.0), *WIPF1* (0.6) | 1 | 64 |

S, Gene-disease association score; S_1_ ≥ 0.3; 0.1 ≤ S_2_ < 0.3; S_3_ < 0.1
