# Supplementary material for: Gene- and Disease-Based Expansion of the Knowledge on Inborn Errors of Immunity
Source: Front Immunol. 2019 Oct 21;10:2475. doi: 10.3389/fimmu.2019.02475 (PMC6816315; doi:10.3389/fimmu.2019.02475)
Supplement: Supplementary file 5 [file Data_Sheet_1.docx]

**Supplementary Table and Figure Legends**

**Supplementary Table 1.** Disease matching in the IUIS, OMIM and ORPHANET databases.

**Supplementary Table 4.** The results of the GWAS Catalog 2019 gene-set enrichment analysis.

**Supplementary Figure 1.** The distribution of top ten HPO terms mapped to the top-level HPO categories: **(A)** Blood and blood-forming tissues, **(B)** Digestive System, **(C)** Immunology, **(D)** Nervous System, **(E)** Cardiovascular, **(F)** Skeletal system, **(G)** Skin, Hair, and Nails, **(H)** Eye, **(I)** Genitourinary System, **(J)** Head and neck.

**Supplementary Figure 2.** DisGeNET variant-disease associations found for the genes of inborn errors of immunity. **(A)** The distribution of genome alternative variant allele frequency (AF_GENOME). **(B)** The distribution of variant-disease association score (vda). **(C)** Scatterplot of genome alternative variant allele frequency and variant-disease association score.
