# Supplementary figures and images for: Gene- and Disease-Based Expansion of the Knowledge on Inborn Errors of Immunity
Source: Front Immunol. 2019 Oct 21;10:2475. doi: 10.3389/fimmu.2019.02475 (PMC6816315; doi:10.3389/fimmu.2019.02475)

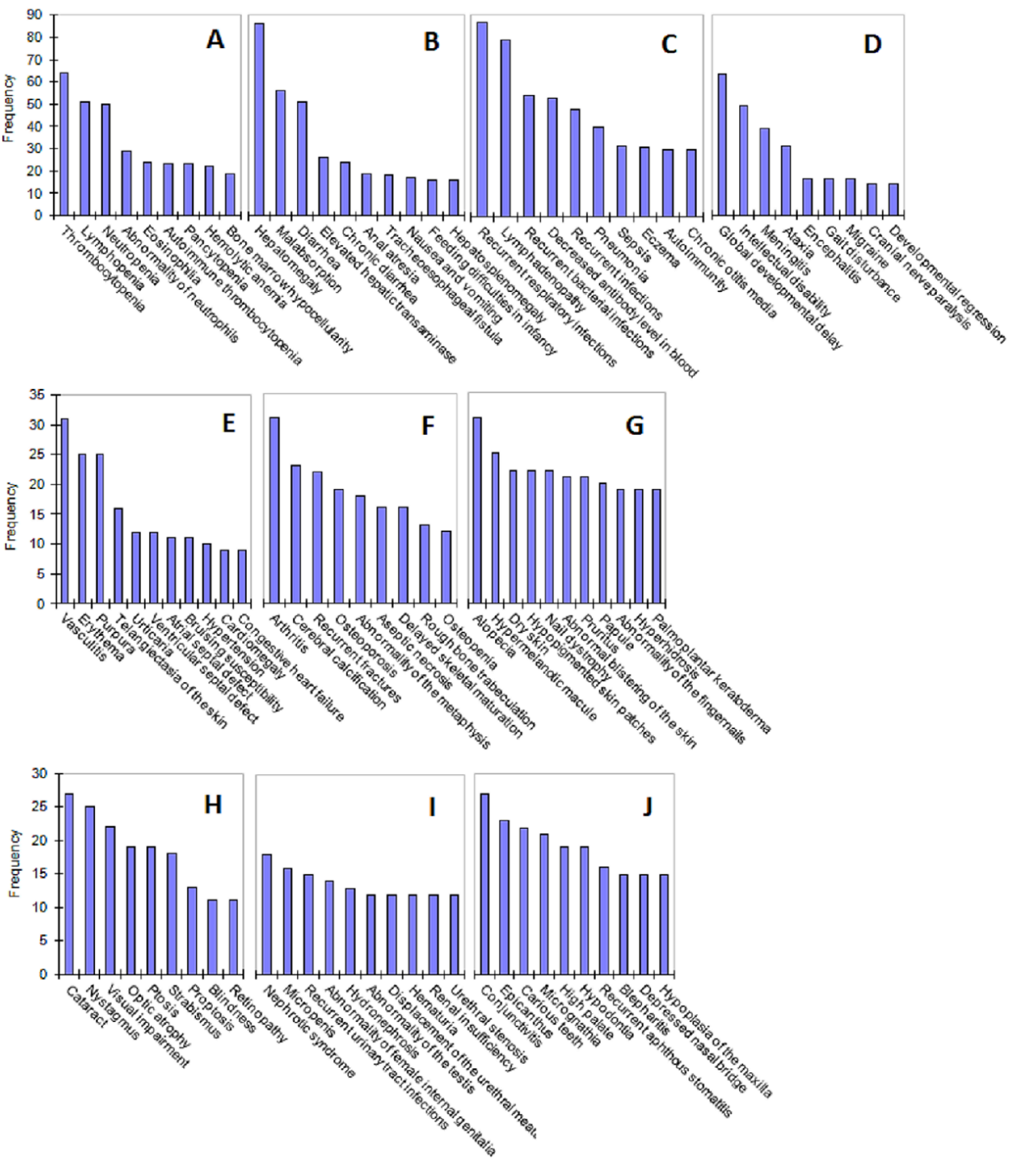

Supplement: Supplementary file 6 [file Image_1.TIF]

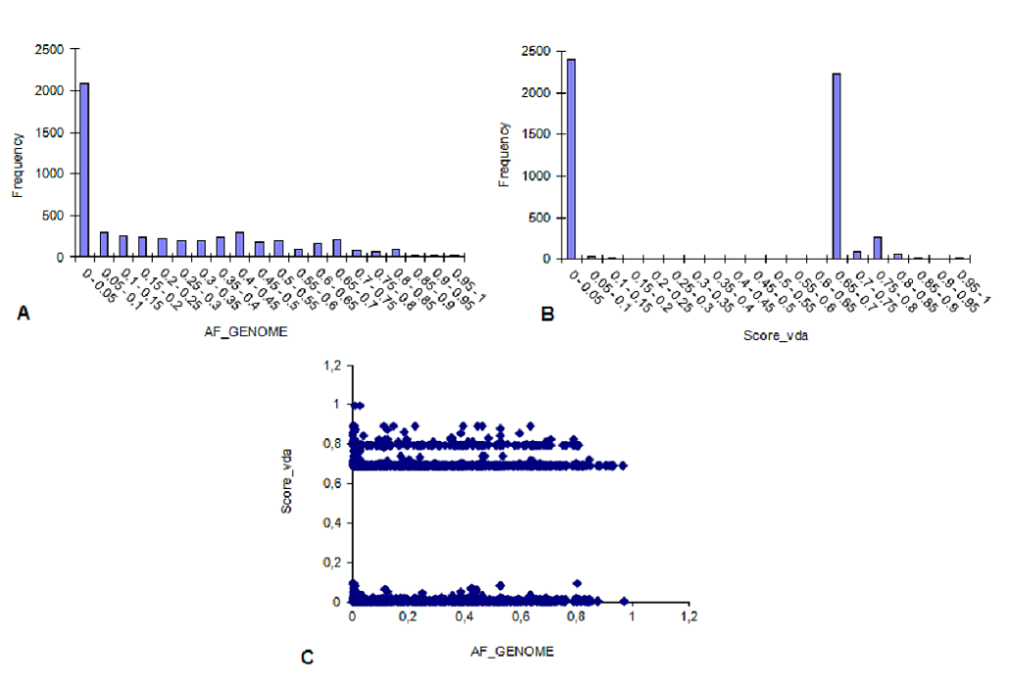

Supplement: Supplementary file 7 [file Image_2.TIF]
